# Innovative digital tools for new trends in teaching and assessment methods in medical and dental education

**DOI:** 10.3352/jeehp.2021.18.13

**Published:** 2021-06-29

**Authors:** Jung-Chul Park, Hyuk-Jae Edward Kwon, Chul Woon Chung

**Affiliations:** 1 Department of Periodontology, Dankook University College of Dentistry, Cheonan, Korea; 2 Department of Oral Biology, School of Dental Medicine, University at Buffalo, State University of New York, Buffalo, NY, USA; 3 Department of General Surgery, International St. Mary’s Hospital, Catholic Kwandong University College of Medicine, Incheon, Korea; Hallym University, Korea

**Keywords:** Augmented reality, Computer-assisted instruction, Digital technology, Patient simulation, Virtual reality

## Abstract

With the goal of providing optimal care to patients, student-centered active learning and the development of clinical competency have become vital components of the education of future physicians capable of sustainably coping with future challenges. However, the shape of future medicine is dramatically changing based on advances in information and communication technology, and the current classroom model seems to have difficulties in fully preparing students for the future of medicine. New trends in teaching and assessment methods include computer-aided instruction, virtual patients, augmented reality, human patient simulations, and virtual reality for the assessment of students’ competency. The digital technologies introduced in medical and dental education include Google Forms to collect students’ answers, YouTube livestreaming, google art & culture (an online art museum), and choose-your-own-adventure as a story-telling technique. Innovations in digital technology will lead the way toward a revolution in medical and dental education, allowing learning to be individualized, interactive, and efficient.

## Introduction

### Background/rationale

As we take stock of the global changes caused by the coronavirus disease 2019 (COVID-19) pandemic, it is clear that the world has radically changed, and new rules, technologies, and institutions are now leading to further transformations. Meanwhile, remarkable advances in digital technologies such as artificial intelligence, deep learning, machine learning, robotic surgery, telemedicine, big data, and next-generation sequencing are now common topics in the medical field. Furthermore, specialized educational content that was previously limited to settings such as medical schools is now being freely released on platforms that can be viewed by anyone around the world. World-renowned medical schools including Johns Hopkins University School of Medicine and Harvard Medical School are providing their excellent teaching materials through YouTube, massive open online courses (MOOCs), or online school platforms. Even specialized training courses in degree programs can be completed fully online [1]. The recognition of limited and specialized education in the medical school context has faded from its old values of authority and scarcity, to the point that it is now regarded as only the minimum qualification for taking the national board examination after one has completed a certain number of school days and credits. In response to these challenges, medical education must now be changed. The William Flora Hewlett Foundation cited the abilities to communicate effectively and collaborate harmoniously, to think critically, to think creatively, to develop academic mindsets, and to learn how to learn. If future medical personnel are educated with these skillsets in mind, the most competent future medical personnel will be those who are not afraid of new things and learn these skills efficiently and quickly [2]. However, we cannot help asking ourselves whether we are still providing medical education with only the aim of training prospective medical personnel who could pass the national licensing examination and placing them into patient care settings. It is also questionable whether education is being properly provided in preparation for further changes in medicine.

### Objectives

In light of the above considerations, we would like to explore experiences of using new educational tools and to think about innovative medical education. Specifically, new trends of teaching and assessment methods are discussed. Next, new tools are presented, including Google Forms to collect students’ answers, YouTube as a new platform in medical education, google art & culture as an online art museum, and choose your own adventure (CYOA) as a story-telling technique. This study may serve as a useful source of information for medical teachers to adopt in the education field.

## New trends in medical education; teaching, and learning

There is no disagreement that in order to produce qualified clinical doctors, it is necessary to conduct clinical education as much as possible. Currently, many clinical lectures engage students through various media, especially photographs and videos, and several educational methods that enhance immersion using recent technologies are also emerging.

### Computer-aided instruction (computer-assisted learning)

This learning method has the advantage of being able to easily visualize complex procedures, and its principal advantage may be excellent accessibility [3]. In particular, through the widespread use of the internet and various electronic devices, clinical education—which traditionally could only be conducted in the school setting, and especially in practice rooms—can now be carried out at home, on the road, and above all, repeatedly at the pace of learning until the students are fully acquainted with the corresponding material. Wang [4] conducted computer-aided training in pharmacology practice classes for Australian medical school students and then surveyed students to evaluate their understanding through a standardized questionnaire. In their study, 98.7% of students successfully achieved their learning goals with a positive result [4]. Of course, in the early days of implementing this learning method, substantial costs are expected to be incurred in order to create this content, but once a certain amount of content is secured, only minimal maintenance costs are required. Of course, this method is not perfect. Above all, since content is provided on a screen using a digital device, it is insufficient to replace practical practice. Moreover, depending on the level of technology, there are cases in which the resolution is insufficient or the reality is poorly presented.

### Virtual patients

This is a form of education that has not been used by many medical schools after it was first proposed by Harless et al. [5] in 1971. The main reason for this is that it is expensive to build such a system, and maintaining it is also not straightforward. Nevertheless, an advantage of this type of education is that customized education is easily available and that students can easily engage in learning through this method without special preparation. The definition of a virtual patient proposed by the American Association for Medical Colleges is a special type of computer program capable of implementing a real patient clinical scenario; it is said that learners can imitate their role as healthcare providers by acquiring a patient’s medical history, conducting a physical examination, and making diagnosis and treatment decisions. Currently, robotic systems involving manikins are common, but thanks to recent technological advances, virtual reality tools are now being used in many cases. The limitations of closed head-mount devices and the provision of equipment are the biggest obstacles, but it is thought that further technological advances will enable these obstacles to be overcome.

### Augmented reality

The equipment called Hololens from Microsoft (Microsoft Corp., Redmond, WA, USA), in the form of augmented reality, provides the unique experience of seeing a real patient and acquiring related medical information, including images and charts. In a study by Pratt et al. [6] in 2018, images could be controlled by voice through the Hololens device, and a case of reconstructive surgery was reported using this technique. According to the results of a systematic review comparing the virtual-patient education method with the existing traditional education method, it was found that the use of virtual patients was more effective in helping students to acquire clinical skills and knowledge compared to existing education methods [7].

### Human patient simulation

This is the most traditional medical education format, and it provides an environment suitable for educational goals using mannikins or models, in which students receive training to cope with various situations models. Although this is a traditional educational method, it has recently become possible to produce a model of the same shape as the patient’s organs using 3-dimensional (3D) imaging techniques and 3D printing, so it has begun to receive attention once again. Most of all, this simulation method enables repetitive practice of specific skills using only partial organs rather than the patient’s entire body, so that immersion is excellent, active experiences are possible, and emotional and sensory learning can be maximized. In addition, it is an excellent learning method for transferring the content of basic medicine to clinical practice. However, in order to provide such equipment, there are problems of cost and space, and it has been pointed out that it may be difficult to provide equipment depending on the number of students.

## New trends in medical education: assessment

Evaluation methods are the field showing the most changes in light of developments in educational technology. Most evaluation methods used in existing medical education are paper-written, oral, or practical tests, and the biggest problem pointed out regarding paper-written tests is that it is difficult to provide sufficient feedback to students due to the structural factor of the overwhelmingly small number of evaluators compared to the number of students.

Many medical school professors experience difficulties simply in preparing exam questions, scoring them, and notifying students of their grades in time. In addition, as most tests involve checking a large amount of knowledge within a short time through simple memorization, short-answer and multiple-choice questions predominate. However, it seems that knowledge of ‘why’ and ‘how’ is much more appropriate for real-world problem solving than ‘what’ itself. For example, asking “what is the criterion for hypertension” could be a very good example of testable knowledge, but that knowledge itself may not be very meaningful in the real world, and if the criterion used to diagnose hypertension changes, the knowledge becomes irrelevant. However, if there is a situation in which a virtual patient takes blood pressure pills regularly, exercises hard, and has recently adjusted his weight, but his blood pressure has not improved at all, that would be a real-world problem that would require the student to think about ‘why’ such a problem arises and ‘how’ to solve the problem, not ‘what’ is the criterion for defining hypertension. Perhaps the true evaluation and preparation for the real world will be helping students to solve real-world situations based on ‘why’ and ‘how,’ not just ‘what.’ On a similar note, the most suitable assessment of students will not be to test whether they know something, but to identify what they do not understand, which must be corrected during the assessment. However, this goal has not been easy to achieve in the traditional assessment format.

## Experiences of using new educational tools

### Google Forms to collect students’ answers

In light of the above considerations, the authors have recently begun to actively utilize educational technology tools, especially Google Forms (https://forms.google.com). Above all, this technology has the advantage that students’ answers are automatically scored and counted in real time, which can substantially save time for the instructor. The learner has the advantage of being able to solve problems and immediately check whether the answer is correct, and also students can review the questions using pre-entered explanations from the instructors to adjust their understanding, which can happen asynchronously at the student’s own pace.

### YouTube

The video platform presented by YouTube contains an overwhelming amounts of videos uploaded by users from all over the world, and users can shoot, edit, and upload directly. Since a large amount of educational content, as well as entertaining content, is uploaded and streamed, people can use YouTube as a tool for learning. The YouTube team also curates and provides learning content (https://www.youtube.com/learning).

**Fig. 1.**
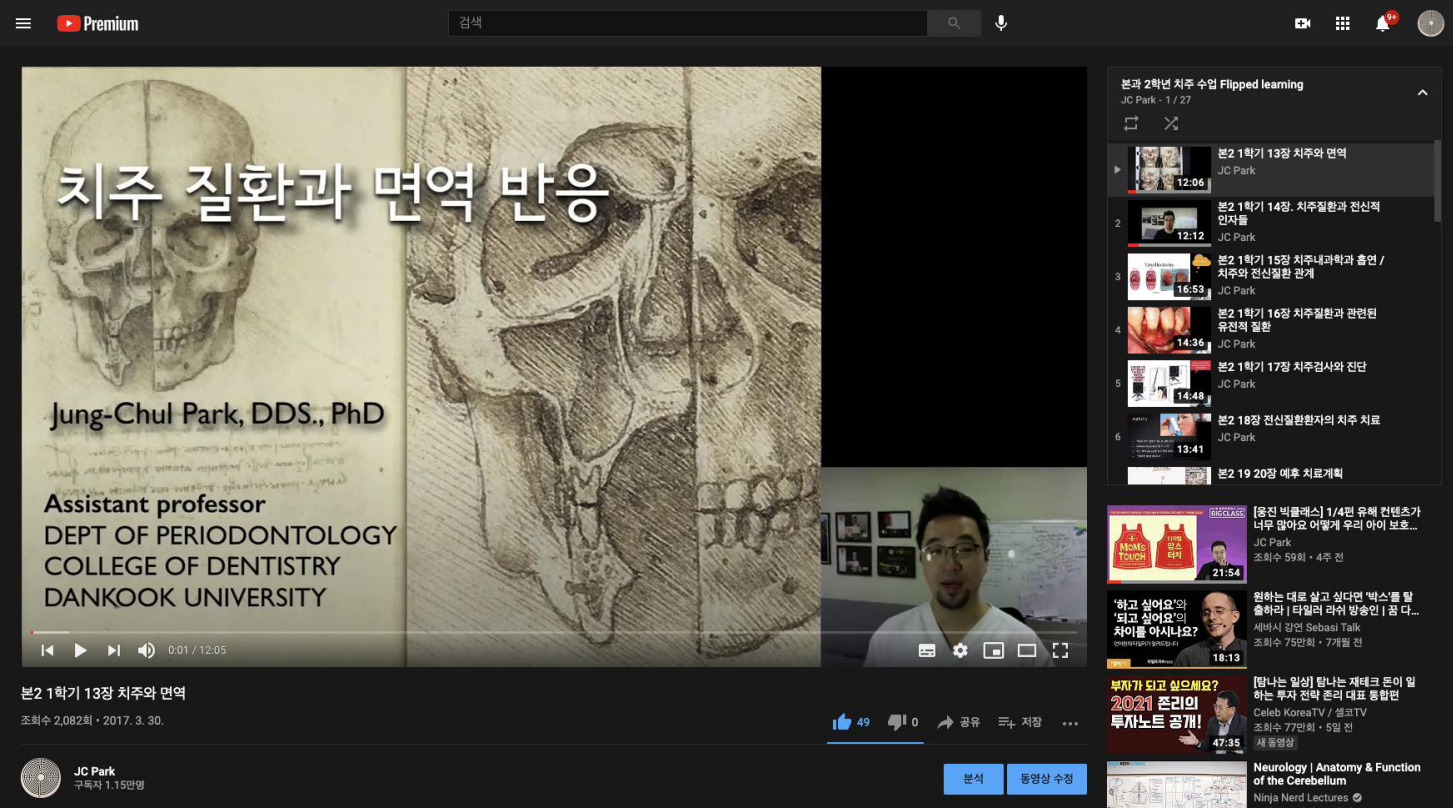
Videos posted on YouTube for the first semester of the second grade of dental school students. Students come to the classroom after previewing the video on YouTube, and in the classroom, various activities such as quizzes, discussions, presentations, and room-escape games are held, available from: https://www.youtube.com/watch?v=ERXWExOzf6Q. (The photo was provided by the authors.)

The author also created a personal channel on YouTube and posts educational materials for flipped learning so that students can watch the material before class (Fig. 1). The biggest advantage of YouTube is that students can easily access YouTube on any device without even signing in, and they are already fully comfortable with how to use the platform. Another important feature of YouTube is the recommended playlists displayed on the top right. The YouTube algorithm automatically select similar content and provides suggested videos alongside. Students can make connections with the original video and acquire profound insights related to materials by watching similar videos from different viewpoints.

**Fig. 2.**
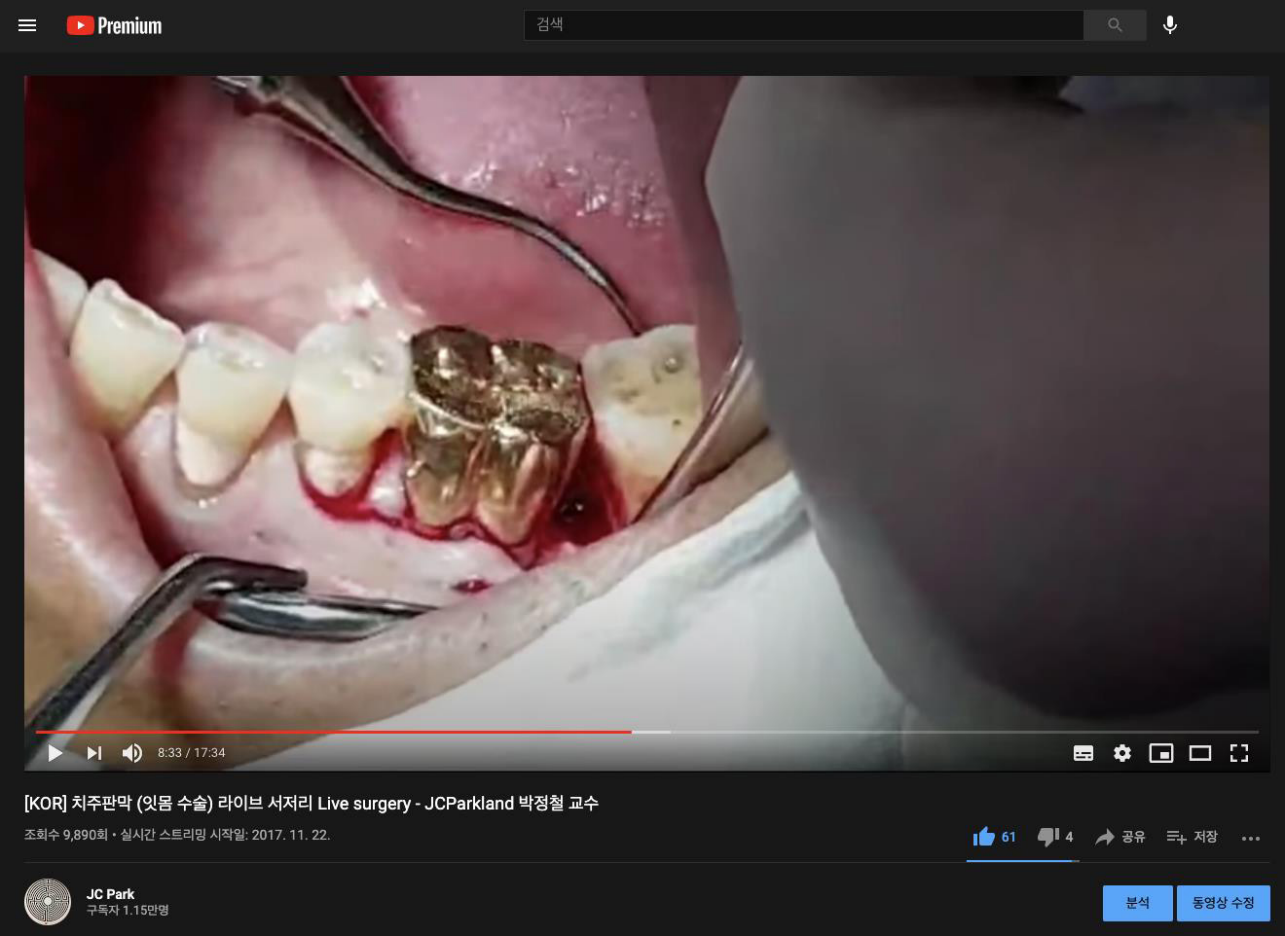
Live surgery scene using the YouTube streaming function. While explaining periodontal flap surgery to second-year dental school students, the class was conducted live at the surgery site, not in the classroom, available from: https://www.youtube.com/watch?v=yromtkKLwdA. (The patient’s prior consent was obtained and caution was taken not to film any personal information of the patient. The photo was provided by the authors.)

In addition, by using the basic streaming function of YouTube, it is possible to provide real-time interactive classes to remote students. For instance, the author conducts live surgery remotely broadcasted using the YouTube live streaming feature to students in the classroom for the introductory lesson for second-year dental school students every year (Fig. 2). This is a very intriguing way to communicate the overall content of surgery to students and to induce interest, and above all, it is thought that using this technique can make the class more meaningful for students who are taking their first steps as clinicians.

However, YouTube live streaming clearly has the disadvantage of being unable to evaluate students’ reactions face-to-face, although students can at least communicate through comments in real time. Fortunately, this technical limitation can be easily overcome by using various types of video conferencing tools, especially Zoom or Google Meet.

**Fig. 3.**
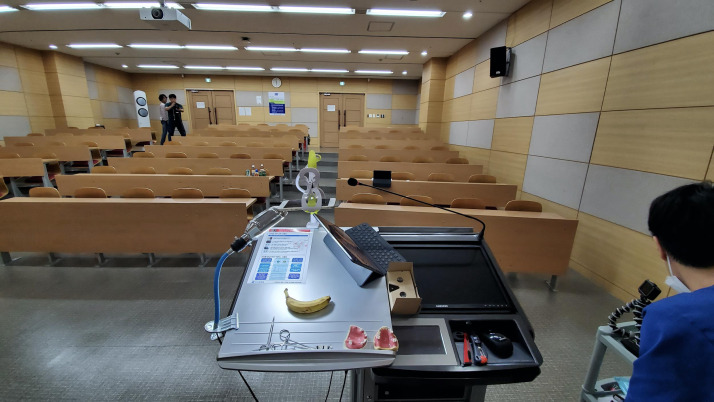
Students gathered using Google Meet, and the author livestreamed the suture techniques online at various angles with a total of 4 cameras using 2 smartphones, 1 tablet, and 1 laptop. At the end of the session, students compared their results with those of other students by showing their results on their own webcams. (The photo was provided by the authors.)

When students could not attend school during the 2020 COVID-19 pandemic period, the author used a streaming application (https://streamyard.com) and multiple mobile devices in order to provide various angles to stream the hands-on suturing lecture (Fig. 3). Students staying at home picked up bananas and suturing instruments beforehand, while maintaining physical distance. The video feed for the demonstration with various angles was livestreamed to the students, who had their own session to practice. At the end of the class, the students were invited to a Google Meet meeting, where they presented their work in turn, and the students had a very productive time evaluating and learning from each other’s work. This episode clearly demonstrated that even a hands-on workshop can be remotely done.

### Google art & culture (an online art museum)

Google art & culture is an online art museum that has formed partnerships with over 1,800 art galleries and museums in 150 countries around the world to scan and upload artworks owned by each institution using Google’s Gigapixel Camera. Armand Trousseau once said that “The worst scientist is he who is not an artist; the worst artist is he who is no scientist” [8]. For physicians, medicine and art cannot be separated from each other. In connection with this, medical education is gradually increasing the frequency of integrating art education. Professor Irwin Braverman of Yale Medical School has argued that through appreciation of artworks, we can gradually develop the ability to observe patients’ diseases [9].

**Fig. 4.**
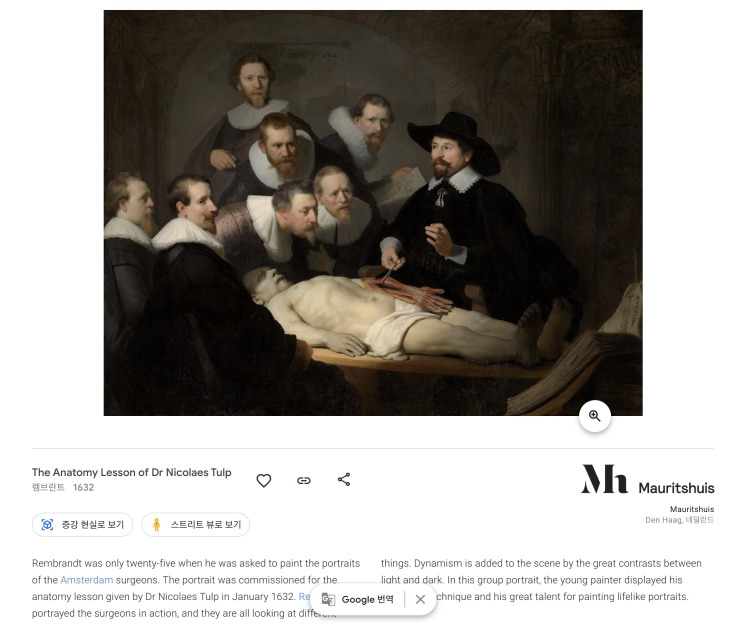
“The anatomy lesson of Dr. Nicholaes Tulp” by Rembrandt (1632) [10].

Among the various works that can be searched on the google art & culture website (https://artsandculture.google.com/), a good example for medical students is an artwork called “The anatomy class of Dr. Nicholaes Tulp” by Rembrandt [10] (Fig. 4). In this artwork, portraits of doctors commissioned by the Dutch Surgeons’ Guild are included. Surprisingly, in the left arm of the cadaver being dissected in the painting, we can find an important error of the anatomy, which has been questioned for a long time regarding the accuracy of the anatomy or the drawing. Using a conventional textbook, it would not be possible to see this point in detail, but one can thoroughly zoom in on Rembrandt’s paintings taken with a gigapixel camera. If one looks closely at the left arm of the body, a mysterious white cord that courses along the ulnar aspect of the cadaver’s carpus and little finger can be noted. This has been considered to be an ulnar nerve variation or artistic error, but in 2007 Jackowe et al. [11] found the same tissue in the actual anatomy of a middle-aged man in his 40s, and concluded that it most likely is the tendon of a variant forearm muscle, an accessory abductor digiti minimi. There are many more similar stories related to medical knowledge infused in artwork, and students can enjoy the content as well as learn from those works.

### Choose your own adventure story-telling technique

CYOA is a story-telling technique commonly found in mystery novels, and refers to a genre in which readers directly intervene in the story at important points to make decisions, and the ending of the book varies according to the decision. Whereas many novels have a single storyline created by the author, the reader in CYOA can actively intervene to create an optimal story and take steps to lead to a happy ending, which can make the process more fun and give a sense of mystery.

In medical education, case-based learning, which utilizes clinical cases to aid teaching, has been demonstrated to be effective in preparing students for clinical practice. If CYOA is applied to such a modality, a very interesting learning process would be feasible. To perform a differential diagnosis, clinicians should keep questioning and ruling out options until there remains only one answer, which is the right disease, and this process is very similar to CYOA. If students can practice making their own CYOA while recording these thought processes in their own CYOA template, they will be able to naturally familiarize themselves with important issues involved in making an accurate diagnosis. Although this approach requires substantial effort from educators, good scenarios will help students to consider various decisions, train them in the differential diagnosis, and help them share the joy of good scenarios with fellow learners.

## Conclusion

Numerous medical schools around the world are recognizing the importance of future-oriented education and are introducing dramatic changes. However, the spread of changes in this process is not easy, and the biggest challenge relates to the burdens or fear of educators themselves. In the process of becoming a medical school faculty member, no one is ever professionally trained in pedagogy—and this situation holds true for medical education in general. It is difficult to bring about innovations in medical education administered in this way. Above all, since most of the tools of future education are digital, this challenge is not easy for professors who feel the technical and psychological burden of the digital transformation. Furthermore, if a professor participates in this challenge to make classes simple and take steps to educate students more effectively, he or she might feel frustrated upon realizing that it is necessary to install new software, learn how to use it, film and edit lecture videos, and respond to online comments. It will not be easy to pursue this journey. Therefore, some colleges have secured the assistance of experts to help and support professors’ difficulties when embarking upon these innovations and changes. Depending on the characteristics of the subject taught by each professor and the professor’s ability to use digital technology, it has been reported that efforts to help professors adjust to this process have been facilitated through a one-on-one matching service.

Of course, huge labor costs are incurred in this part of the transition, but the larger the ship is, the more fuel is consumed to change its direction. At least, many of the tools that the author uses are easy-to-use and provided free of charge. There are many good tools that are even better than these, but the more features there are, the more difficult it is to learn how to use them. Then, the burden on the instructor also increases. There will always be tools and methods that are overwhelming and on the cutting edge of innovation compared to existing educational modalities, but on the contrary, it is also very intriguing to hear that it is possible to considerably surpass many aspects of existing education by using common tools that are very easy and available for anyone to use. Destructive innovation in medical education should be expected in the near future.

## Supplementary materials

Supplement 1. Audio recording of the abstract.
